# Case Series Review on Use of Topical Timolol in the Treatment of Superior Oblique Myokymia

**DOI:** 10.22599/bioj.376

**Published:** 2024-12-02

**Authors:** Victoria Alexis Smerdon, Joanne Adeoye, Alison Rowlands

**Affiliations:** 1 Wirral University Teaching Hospital, UK; 2 University of Liverpool, UK

**Keywords:** superior oblique myokymia, timolol, off label ocular beta blocker

## Abstract

**Introduction:**

The treatment options available for Superior Oblique Myokymia (SOM) previously included surgery and systemic drugs, with the addition of beta blockers from the early 1990’s. This case review aims to examine the efficacy of topical timolol (0.5%) as a treatment for SOM in the short and long term.

**Methods:**

This is a retrospective case series review on a group of patients identified with this condition who have been assessed and treated with topical timolol 0.5% (off-label), within the same district and general hospital over a period of four years.

**Results:**

Six patients were identified from the case review. Five out of the six patients were treated with the use of off label timolol 0.5%. One patient declined treatment due to other systemic conditions and another later discontinued treatment due to change in cardiac status. Two of the patients required increased dosage from OD to BD to achieve success initially. All the patients treated were symptom free at eight weeks and remained symptom free at six months follow-up. None of the patients in the cohort reported significant side effects.

**Conclusion:**

This case series highlights the efficacy of the use of off label timolol 0.5% for the management of SOM with an 80% success rate for those treated with the drug.

## Introduction

Superior Oblique Myokymia (SOM) is a rare condition, characterized by episodes of sudden monocular rapid eye movements of small amplitude that may be vertical, torsional, or oblique. These movements result from involuntary rhythmic contraction of the superior oblique muscle. As a result, patients experience oscillopsia and diplopia, particularly when vision is directed downwards and often precipitated by reading (Lawden, 2018). In a meta-analysis of 116 cases, age at examination ranged from 17–72 years (mean 42 years), 52% of cases were female, and 61% occurred in the right eye (Zhang et al., 2018b).

The aetiology of SOM is unknown. Ephaptic transmission has been proposed as a likely contributor (Zhang et al., 2018b). There are case reports of occurrences associated with compressive lesions or trauma (Hashimoto et al., 2004; Adamec and Habek, 2018). Anecdotal evidence within the literature indicates the condition may be precipitated by stress, excessive alcohol use, and fatigue (Borgman, 2014). Sathyan and Antony (2017) hypothesised a potential photo sensitivity trigger for the condition, as they were able to elicit onset with a flashlight. Lee (1984) identified a link between SOM and mild weakness of the IV cranial nerve.

Literature on the role of imaging in the investigation of these patients is conflicting. Patel and Malhotra (2023) stated that MRI is recommended only for patients who were nonresponsive to medication, as the incidence of brainstem lesions in relation to SOM is low. Others, however, advocate more extensive imaging as a key part of the investigative work up for these patients (Tandon and Oliveria, 2019)

The use of beta blockers in the treatment of this condition was first documented by Tyler (1990) using oral propranolol 10 mg daily. The successful use of topical beta blockers in the treatment of SOM have been reported in single case reviews by Bibby et al. (1994), Borgman (2014), Mickelson et al. (2004), Heaven et al. (1995), and Gupta et al. (2007). Successful use of levobunolol hydrochloride (0.5%) in two patients was reported by Zhang et al. (2018a). There are two main theories for the mechanism of the effect of beta blockers in SOM. The first one involves the direct effect on the nerve versus the peripheral systemic effect on the system supporting the nerve. Zhang et al. (2018a) hypothesised that the improvement effect was due to the systemic ocular absorption through the ocular blood vessels causing membrane stabilisation and improved ephaptic transmission. However, Borgman (2014) proposed a second hypothesis, posing that the impact of the Beta Blocker was on the trochlear nerve ending, which impacted the Superior Oblique tissues, which in turn eliminated the oscillation.

Prior to the usage of topical beta blockers, the main treatment options were surgical; Superior Oblique Myectomy/Tenotomy, which carries a risk of post-operative diplopia (Agarwal and Kushner, 2009) or microvascular decompression of the superior oblique (Samii et al., 1998). Medical management via the use of systemic drugs such as gabapentin memantine and propranolol (Tomsak et al., 2002) have high reported rates of success (up to 80%) (Susac et al., 1973; Williams et al., 2007) but carry a risk of systemic side effects (Patel et al., 2016).

This case review aims to examine the efficacy of topical timolol (0.5%) as a treatment for SOM. This review also aims to highlight the condition as a potential diagnosis in the presence of a mild superior oblique under action, good binocular functions, and visual disturbance, particularly post-reading.

## Methods

Approval for this case series review was provided by the Clinical Governance and Clinical Research Departments of Wirral University Teaching Hospital. Cases for inclusion in the review were identified retrospectively via a data pull from the Trust Electronic Patient Record. All patients with a confirmed diagnosis of SOM over a period of four years were included. Full orthoptic investigation and slit lamp examination were undertaken by an Orthoptist and an Ophthalmologist with expertise in neuro-ophthalmology. Diagnosis of SOM was made in the presence of SO underaction and uni-ocular oscillation, seen either in free space or on slit lamp examination confirmed with the use of cover test and smooth pursuit testing.

All patients underwent cranial MRI prior to treatment to rule out any other causes, and were reviewed for potential contraindications (e.g., cardiac issues, asthma, etc). Patients were offered treatment with timolol 0.5%, counselled as to the off-label use of the medication, and a shared decision to treat was made. Verbal advice was given on instillation and occlusion of the puncta to minimise systemic effects alongside written information on the drug and its actions. Initial dosage was once daily (OD) to the affected eye. This dosage was increased to a maximum of twice per day (BD) if symptoms persisted at the four-week review.

Patients were reviewed after four weeks. Review consisted of orthoptic and ophthalmic examination by a specialist orthoptist. Symptoms were reported subjectively. Successful treatment was defined as a complete elimination of ocular symptoms, namely, no oscillation or diplopia.

## Results

Six patients (Table 1) were identified for case review (four males and two females), with a mean age of 61 (range 47–67). The right eye was the affected eye in all but two of the patients in the sample. All patients reported experiencing visual disturbance and oscillopsia as their main presenting symptom. One patient experienced diplopia and one reported ‘ghosting.’ All patients were diagnosed as having a mild SO (–0.5 to –1) weakness in the same eye that experienced the oscillation. None of the patients in the series displayed any manifest deviation in any position of gaze. None of the patients demonstrated manifest or latent vertical deviation in the primary position. All showed normal MRI’s in keeping with expected age-related findings. None of the age-related changes reported were in the region of the fourth cranial nerve pathway.

**Table 1:** Summary of patients included in the case review.


SEX	AGE AT PRESENTATION	AFFECTED EYE	PATIENT REPORTED PRESENTING SYMPTOM	DOSAGE & DURATION	OUTCOME

Female	61	Right Eye	Rapid oscillation of one eye	0.5% OD Ongoing	Symptom free at 4/52, 6/12 and 12/12 follow up

Female	76	Right Eye	Episodic vertical oscillation/diplopia	N/A	Declined treatment due to risk associated with COPD

Male	54	Left Eye	Oscillation/shimmering vision	0.5% OD Ongoing	Symptom free at 4/52 and 6/12 follow up

Male	67	Right Eye	Episodes of ‘wobbling vision’	0.5% OD 4 weeks; 0.5% BD 4 weeks; Gabapentin 300 mg initially due to be increased to 900 mg over staged period	All treatment discontinued due to change in cardiac status

Male	62	Left Eye	Ghosting/Moving image	0.5% OD 4 weeks 0.5% BD Ongoing	Reduced Symptoms at 4/52 Symptom free after 8/52 and 6/12 follow up

Male	47	Right Eye	Intermittent vertical shaking of vision	0.50% OD 4 weeks 0.5% BD Ongoing	Reduced Symptoms at 4/52. Symptom free at 8 week and 6/12 follow up


Five out of the six patients were treated with the use of timolol 0.5% (off-label). Of the five patients treated four (80%) were successfully symptom free after eight weeks and at six month follow up. Two patients increased dosage from OD to BD to achieve success. None of the patients in the cohort reported any side effects from the medication aside from mild stinging on installation.

Of the two unsuccessfully treated patients within the case review, one patient had a confirmed diagnosis of COPD and decided that the risk of ocular beta blockers did not outweigh the potential benefit in terms of their symptoms. In view of the identified co-morbidities, this patient declined pharmacologic treatment and managed their symptoms conservatively. With regard to the second unsuccessful patient, their symptoms persisted despite an increase in dosage of the off label timolol, so they were swapped to oral gabapentin after eight weeks. This patient has subsequently developed heart issues and has been withdrawn from all ocular related medicines, whilst under investigation by cardiology. The cardiology issues were identified as not being related to the initial use of ocular beta blockers.

## Discussion

Due to the rarity of SOM, the literature often features single or small case cohorts with just 116 cases published since SOM was first described in 1906 (Zhang et al., 2018b). Only a small number of studies have reported on the use of topical beta blockers, many of which are single case studies (Bibby et al., 1994; Borgman, 2014; Heaven et al., 1995; Gupta et al., 2007) with one report of two cases (Zhang et al., 2018a).

The patients in our cohort presented at a significantly older mean age than those included in Zhang et al.’s (2018b) meta-analysis (62 vs. 42 years). The cases in this series supported the evidence of a mild right eye preponderance. A point of interest with all six patients was that symptom onset followed periods of high stress or illness. This supports the literature hypothesis discussed around precipitating factors mentioned previously. In line with the findings of previous reports of successful treatment with Beta Blockers, all patients who continued on the treatment of 0.5% timolol experienced successful resolution of symptoms without side effects, further supporting the case for use of topical beta blockers over the use of other oral medications that report similar success rates (Williams et al., 2007) but carry a significantly greater risk of systemic side effects.

## Conclusion

Although a small cohort, this is the largest published case series highlighting the efficacy of the use of off label timolol 0.5% for the management of SOM with a high success rate (80%) for those treated with the drug. Clinicians should be aware of the off-label nature of its use and its potential effectiveness in managing symptoms and be able to counsel patients appropriately.

Given the apparent association with stress or recent illness, clinicians should remember to include detailed questions around mental as well as physical wellbeing as part of routine questioning. Patients reporting any issues affecting their wellbeing should be directed to appropriate resources and support in line with public health advice.

This case series serves as a significant contribution to the sparse evidence base for this treatment approach. The encouraging results represent a low risk/high reward option for management. As SOM is a relatively rare condition, it is difficult to identify large numbers of patients for prospective interventional study. For future studies, a multicentre design may be necessary to further examine treatment response or predictors of success.

## Ethics and Consent

This case review was initially undertaken as an audit of clinical practice and outcomes within the Trust and as such has been fully anonymised data from the beginning. This was given ethical approval to be undertaken by the Trusts clinical governance department at both a departmental and divisional level. The article was also reviewed by our research team and Caldicott guardian and was deemed not to be true research and did not thus meet the need for additional ethical approval prior to submission for publication.
